# Integrating herbivore assemblages and woody plant cover in an African savanna to reveal how herbivores respond to ecosystem management

**DOI:** 10.1371/journal.pone.0273917

**Published:** 2022-08-31

**Authors:** Melissa H. Schmitt, Keenan Stears, Mary K. Donovan, Deron E. Burkepile, Dave I. Thompson

**Affiliations:** 1 Department of Ecology, Evolution and Marine Biology, University of California Santa Barbara, Santa Barbara, California, United States of America; 2 South African Environmental Observation Network, Ndlovu Node, Scientific Services, Kruger National Park, Phalaborwa, South Africa; 3 School of Geographical Sciences and Urban Planning and Center for Global Discovery and Conservation Science, Arizona State University, Tempe, Arizona, United States of America; 4 Marine Science Institute, University of California Santa Barbara, Santa Barbara, California, United States of America; 5 School of Geography, Archaeology and Environmental Studies, University of the Witwatersrand, Johannesburg, South Africa; Bowling Green State University, UNITED STATES

## Abstract

African savannas are experiencing anthropogenically-induced stressors that are accelerating the increase of woody vegetation cover. To combat this, land managers frequently implement large-scale clearing of trees, which can have a cascading influence on mammalian herbivores. Studies rarely focus on how differences in woody cover influence the herbivore assemblage, making it difficult to assess how aggressive measures, or the lack of management, to counteract increasing woody cover affect the local composition and biodiversity of herbivores. We address this knowledge gap by applying a model-based clustering approach to field observations from MalaMala Game Reserve, South Africa to identify multiple herbivore–vegetation ‘configurations,’ defined as unique sets of herbivore assemblages (i.e., groups of herbivores) associated with differing woody plant covers. Our approach delineated how tree-clearing influences the distribution and abundance of the herbivore community in relation to surrounding savanna areas, which represent a natural mosaic of varying woody cover. Regardless of season, both intensively managed areas cleared of trees and unmanaged areas with high tree cover contained configurations that had depauperate assemblages of herbivores (low species richness, low abundance). By contrast, habitats with intermediate cover of woody vegetation had much higher richness and abundance. These results have substantial implications for managing African savannas in a rapidly changing climate.

## Introduction

Savannas cover approximately 50% of the land surface of the African continent [1]. This biome encompasses diverse ecosystems that provide goods and services to human populations as well as critical resources for a diverse set of mammalian herbivores [2]. Savannas are characterized by the co-dominance of trees and grasses, and span a natural woody gradient from densely wooded Miombo woodlands to open systems with widely scattered trees like the Serengeti-Maasai Mara ecosystem [1]. The spatially and temporally variable co-dominance of trees and grasses in savanna systems is influenced by a complex set of interacting biotic and abiotic factors including geology, fire, precipitation, and herbivory [3–7]. Synergistically, these factors create the structural heterogeneity that is inherent to savanna systems.

Woody plant cover is a key factor that influences the distribution of mammalian herbivores across the landscape because it synergistically influences both top-down (i.e., predation risk) and bottom-up (i.e., food availability/quality) processes [8–10]. Specifically, in African savannas, herbivores perceive habitats with low woody plant cover to be less risky because of increased visibility [11]. By contrast, increasing woody plant cover is often associated with increased perceived predation risk [12], most likely due to increased ambush opportunities and reduced visibility. However, woody vegetation can also influence food availability and quality for herbivores. Woody vegetation provides browse for browsing species (e.g., kudu, *Tragelaphus strepsiceros*, and giraffe, *Giraffa camelopardalis*) as well as influences soil properties that can affect the quality and quantity of the herbaceous layer for grazing species (e.g., zebra, *Equus quagga*, and wildebeest, *Connochaetes taurinus*) [13]. Ultimately, herbivore habitat use is influenced by the trade-off between forage acquisition and predation risk [14, 15]. However, the relative importance of top-down and bottom-up processes in influencing this trade-off are context dependent and can vary both spatially and temporally and are further influenced by herbivore body size, feeding guild (i.e., grazers, browsers, and mixed-feeders), and predator avoidance strategies [16–20]. Because the strengths of top-down and bottom-up processes are context dependent, and the trade-off between forage acquisition and predation risk is modulated by woody plant cover, any change in woody plant cover can greatly impact the species composition of the herbivore community [see 10].

Understanding the response of the herbivore community to a woody plant gradient is critically important, especially in the context of global change. For example, African savannas and savannas globally are experiencing shifts towards woody plant dominance via woody thickening (i.e., increases in woody plant density in already-wooded areas) and woody encroachment (i.e., invasion of woody plants in non-wooded areas) [21, 22]. To combat increases in woody plant cover, land managers are implementing extensive habitat management practices in savannas. One approach that is used globally is large-scale mechanical tree-removal to combat increased woody plant cover and promote the productivity of the herbaceous layer [23–26]. Consequently, savanna systems are becoming more open in some areas due to tree removal, and more densely wooded in others due to unmanaged woody encroachment, often resulting in the loss of intermediate, structurally heterogenous habitats across the landscape. Because of the importance of woody plant cover in modulating herbivore distributions, a number of studies have assessed how herbivores may respond to changes in woody plant cover. However, the majority of these studies focus on small-scale controlled experiments [e.g., 25] and/or only explore extreme scenarios of woody plant cover [e.g., open habitats versus densely wooded habitats; 24] and have not explored how herbivores respond to a woody plant gradient. This hampers our ability to predict future effects of increasing woody plant cover in this ecosystem [10].

Here, we leverage large-scale tree clearings to understand how management approaches that control increasing woody cover influence the distribution, abundance, and diversity of the herbivore community. These clearings occur within an African savanna that is characterized by a natural gradient of tree densities, including areas that have experienced woody thickening. This allows assessment of the response of herbivores along a woody plant cover gradient and provides a unique opportunity to address the effects of this widespread management action on herbivore assemblages (i.e., group of species occurring in the same area). We identified unique configurations consisting of herbivore assemblages associated with particular levels of woody plant cover and then used these to explore how woody plant cover influences patterns of herbivore species richness and abundance. We predicted that woody plant cover would structure herbivore assemblages, however, the exact responses of herbivores to woody cover would be modulated by their feeding guild. Specifically, an increase in woody plant cover should favor browsing species while negatively impacting grazing species. Moreover, because of seasonal effects on the resource acquisition-predation risk tradeoff, we predicted that herbivore responses would vary across the wet and dry seasons with herbivores shifting towards woodier areas in the dry season. Finally, using our findings, we identify management approaches that could either facilitate or suppress the diversity and abundance of the savanna herbivore communities.

## Materials and methods

### Study site

We conducted our study in the MalaMala Game Reserve (13,300 ha) within the Sabi Sands Wildtuin–MalaMala Complex, which forms part of the Greater Kruger National Park, South Africa. The study site is unfenced and is bordered by the Kruger National Park to the east and the Sabi Sands Wildtuin to the north, south, and west. The Sand River is the major source of water. There are a number of dams and pans scattered throughout the study site, however, these do not receive artificial water provisioning during the dry season. MalaMala Game Reserve contains a diverse large predator guild including ambush predators such as lion (*Panthera leo*) and leopard (*Panthera pardus*) as well as various cursorial predators including cheetah (*Acinonyx jubatus*), African wild dog (*Lycaon pictus*), and spotted hyena (*Crocuta crocuta*). Additionally, our study site has a diverse herbivore guild (see below).

The vegetation is characterized by a mixed combretum/terminalia woodland [27], with the most abundant species including: *Combretum apiculatum*, *C*. *zeyheri*, *C*. *hereroense*, *Terminalia sericea*, *Senegalia nigrecens*, and *Euclea divinorum*; all of these species occur in the habitats identified in our configurations. In response to increasing woody plant cover, land managers began a widespread, non-selective, woody vegetation (i.e., trees and shrubs) clearing campaign 50–60 years ago (i.e., 1960s–1970s), creating large (30–50 ha) swaths of human-made, artificial grassland [28]. These cleared areas are not thin, linear strips along roads, but rather extend ~200 m away from roads, creating substantial habitat patches. The large size of the tree-cleared areas is congruent with the scale of the decision-making of herbivores in savanna landscapes (i.e. selection for habitat patches that represent numerous behavioral opportunities associated with vegetation patterns) [29]. In these areas, only large trees (>5m height) were retained and, after clearing, tree cover densities ranged from 0–5 trees/50 m^2^. The remaining tree species in these areas are the same species found in the adjacent mosaic of savanna vegetation. The tree-cleared areas have been maintained by annual mowing creating open, artificial grasslands that occur within a natural mosaic of woody plant cover ranging from open savannas to woody thickets.

### Quantifying patterns of herbivore abundance across a woody gradient

We quantified patterns of abundance of 12 species of mammalian herbivores: African buffalo (*Syncerus caffer*), common duiker (*Sylvicapra grimmia*), African elephant (*Loxodonta africana*), giraffe, impala (*Aepyceros melampus*), kudu, nyala (*T*. *angasii*), steenbok (*Raphicerus campestris*), warthog (*Phacochoerus africanus*), blue wildebeest, white rhinoceros (*Ceratotherium simum*), and plains zebra. These species fall into one of three feeding guilds: browser (eats primarily woody vegetation), grazer (eats primarily grasses), or mixed-feeder (eats both woody vegetation and grasses) (see S1 Table).

To assess the abundance and habitat associations of the herbivores, we conducted standardized daily driving transects (20 or 40 km per day) during the wet (January–March, n = 33 days) and dry (July–September, n = 47 days) seasons of 2018, following the guidelines of Caro [30]. Such transects are a common approach to quantifying herbivore habitat use associations [8, 30–32]. These transects were conducted from established dirt roads, which run through representative habitats within the study area, *as per* Caro [30]. The transects spanned a woody cover gradient that ranged from areas cleared of woody plants (i.e., active management against woody thickening) to areas with high woody plant densities (i.e., passive management of woody thickening). For our transects, we drove at speeds <20 km/h in the early morning (0800–1000 hr) and late afternoons (1500–1700 hr) and conducted them >1 km away from the river and avoided dams and pans where possible. This limited the potential confounding factors of time of day, which influences thermoregulatory behaviors and perceived predation risk, as well as surface water on herbivore habitat association patterns. The animals at our study site are habituated to vehicles and do not flee when they encounter vehicles. While vegetation structure can limit the ability of observers to detect small, elusive herbivores [31], the pattern of low herbivore abundance in densely wooded habitats is well documented in African savannas [10] and is frequently attributed to increased predation risk [12, 33, 34]. Thus, observed low species abundances are not necessarily linked with low detectability. To confirm that our sampling procedure was robust for detection of species abundances, we conducted dung surveys. We sampled 56 plots (25x25 m each) along a woody plant cover gradient spanning from tree cleared open areas to dense encroached habitats. Within each plot, we identified and enumerated all dung present. Ultimately, we found qualitatively similar unimodal patterns with low richness and abundance at either end of the woody cover gradient, and highest use and richness at intermediate levels of woody plant cover (see Results below and S1 Fig). Thus, our reported patterns of herbivore abundance are not likely driven by detectability, especially given that dung counts are a comparable method to determine herbivore habitat use [see also 35].

The same two observers enumerated all herbivore species encountered within 50 m of the road, which is within the established detection range for the majority of large mammals, as per Caro [31] (wet season n = 33 days, 9,961 individuals from 1,527 herds or sightings; dry season n = 47 days, 9,074 individuals from 1,289 herds or sightings). For each herbivore encountered, we identified the species, counted the number of individuals in its herd if applicable, and assessed percentage woody plant cover. We visually quantified the percentage woody plant cover within a ~50 m radius around each individual or herd, which ensured that the categorization of the area around each herd or individual reflected the larger habitat and did not reflect a finer-scale estimate of a microhabitat within the larger habitat. Additionally, the proportional availability of habitats with different woody plant cover along our 40 km transect was quantified by visual assessment of the percentage woody plant cover on either side of the road at 100 m intervals (n = 800 points). At each point, we classified the habitat into one of six key types found in African savannas: (1) artificial grassland with <5% woody cover, (2) open canopy savanna with ~15% woody cover, (3) semi-open canopy savanna with ~30% woody cover, (4) woody savanna with ~50% woody cover, (5) closed-canopy woody savanna with ~75% woody cover, and (6) thicket with >75% woody cover (see S2 Fig). To link percent woody plant cover with woody plant density, we enumerated all woody vegetation (>1.5 m in height) in 25 x 25 m plots in areas representative of five of the six habitat types (N = 55). For the thicket habitat, we used the estimate of woody plant density in Belsky [36]. Our estimates of woody plant density in our savanna system are within the range of other published studies conducted in African savannas. For example, Ben-Shahar [37] reports as few as 0 trees/ha in tree-cleared areas that are equivalent to our artificial grasslands. Barot et al. [38] reports tree densities of ~80 trees/ha for open savannas (the equivalent of our open-canopy savanna), ~90–145 trees/ha for tree savannas (the equivalent of our semi-open canopy savanna), and ~600 trees/ha for savanna woodlands (the equivalent of our closed-canopy woody savanna). Similarly, Wakeling et al. [39] estimate that 400 adult trees/ha would create a savanna woodland (the equivalent of our woody savanna). To account for differences in sampling effort across replicates (days), we weighted the observed abundance of each herbivore species by the unit effort (i.e., distance traveled each day). Finally, given that we were primarily interested in the large-scale physiognomy of a habitat in which the herbivore community occurs [40], we did not focus on elucidating habitat variables, such as patch quality and distance to ambush sites, which influence patch selection of herbivores at smaller scales [14].

Because diel cycles can influence patterns of herbivore habitat use [e.g., 41], we corroborated our observed patterns of both abundance and richness across a woody plant cover gradient that were collected during daylight hours via driving transects with dung counts (described above). Dung counts are conducted within a set sampling area and represent a time-averaged (i.e., both day and night) index of animal use. We found qualitatively similar unimodal patterns with low richness and abundance at either end of the woody cover gradient, and highest use and richness at intermediate levels of woody plant cover (see Results below and S1 Fig). Thus, our reported patterns are not driven by the diel cycle. Because all aspects of this work were observational, no ethical clearance was required. Permission to conduct this research was provided by the land owner.

### Data analyses to identify configurations and seasonal patterns

Similar to Hempson *et al*. [42], who used a clustering approach to determine which environmental variables influenced herbivore assemblages at a continent scale, we used a clustering approach to explore the relationship between the mammalian herbivore assemblage and woody plant cover. To do this, we identified configurations representing various subsets of the overall herbivore community and their associated woody plant cover using a model-based clustering approach with the *mclust* package in R [43]. This framework uses three strategies for defining clusters (i.e., configurations): (1) initialization of the model with model-based hierarchical clustering, (2) maximum likelihood estimation with the expectation-maximization algorithm, and (3) model selection and the number of clusters that are approximated with Bayes factors and Bayesian Information Criterion [44]. Thus, the cluster analysis is based on a probability model where each cluster is a mixture of multivariate normal distributions composed of the densities of each component, and each observation is assigned to a cluster based on the probability of membership given the observation, as per Donovan *et al*. [45]. The 12 herbivore species and percent woody cover were the model inputs. Inputs were square-root transformed to standardize all variables before clustering. Outputs were model-identified configurations that represent unique herbivore assemblages and their occurrence along the woody plant cover gradient. In this framework, the woody plant cover associated with each herbivore assemblage to create a configuration, was based on the mean woody plant cover where the clustering had the greatest probability of occurrence. We ran a separate clustering analysis for each season.

The availability of habitats with different levels of woody plant cover can influence herbivore habitat use. Thus, to assess whether our configurations were influenced by habitat availability, as well as make them more meaningful, we linked percentage woody plant cover associated with the configurations with six key habitat types found in African savannas (as described above) based on their average woody plant cover [e.g., 46]. The highly-managed artificial grasslands and the dense woody thicket habitats fall within the typical ranges of savanna woody vegetation cover (see above), however, their prevalence across savanna landscapes are increasing due to anthropogenic disturbances. The classifications of the six habitat types are consistent across seasons.

To investigate whether herbivore species richness and abundance varied with woody plant cover, we used generalized linear models (Gamma distribution with a log link function) to compare total herbivore richness and total abundances across configurations in each season. Model contrasts and confidence intervals were calculated with Tukey’s Honest Significant Difference Tests. In addition, herbivores were grouped into their respective feeding guilds (i.e., grazer, mixed-feeder, browser) and the seasonal abundance of each feeding guild category across the different model-generated configurations was analyzed using generalized linear models (Compound Poisson distribution with a log link function from the Tweedie family of distributions) using the *statmod* package in R, following Dunn and Smythe [47]. We used the Tweedie family of distributions because our data consisted of both zeros and positive values [48]. To reduce the Type 1 error probability, we used Holm’s [49] correction of α for sequential analyses of the same null hypothesis. All data and code used in the manuscript are available from the Zenodo Repository [50].

## Results

### Identifying configurations of herbivores and woody plant cover

**Fig 1:**
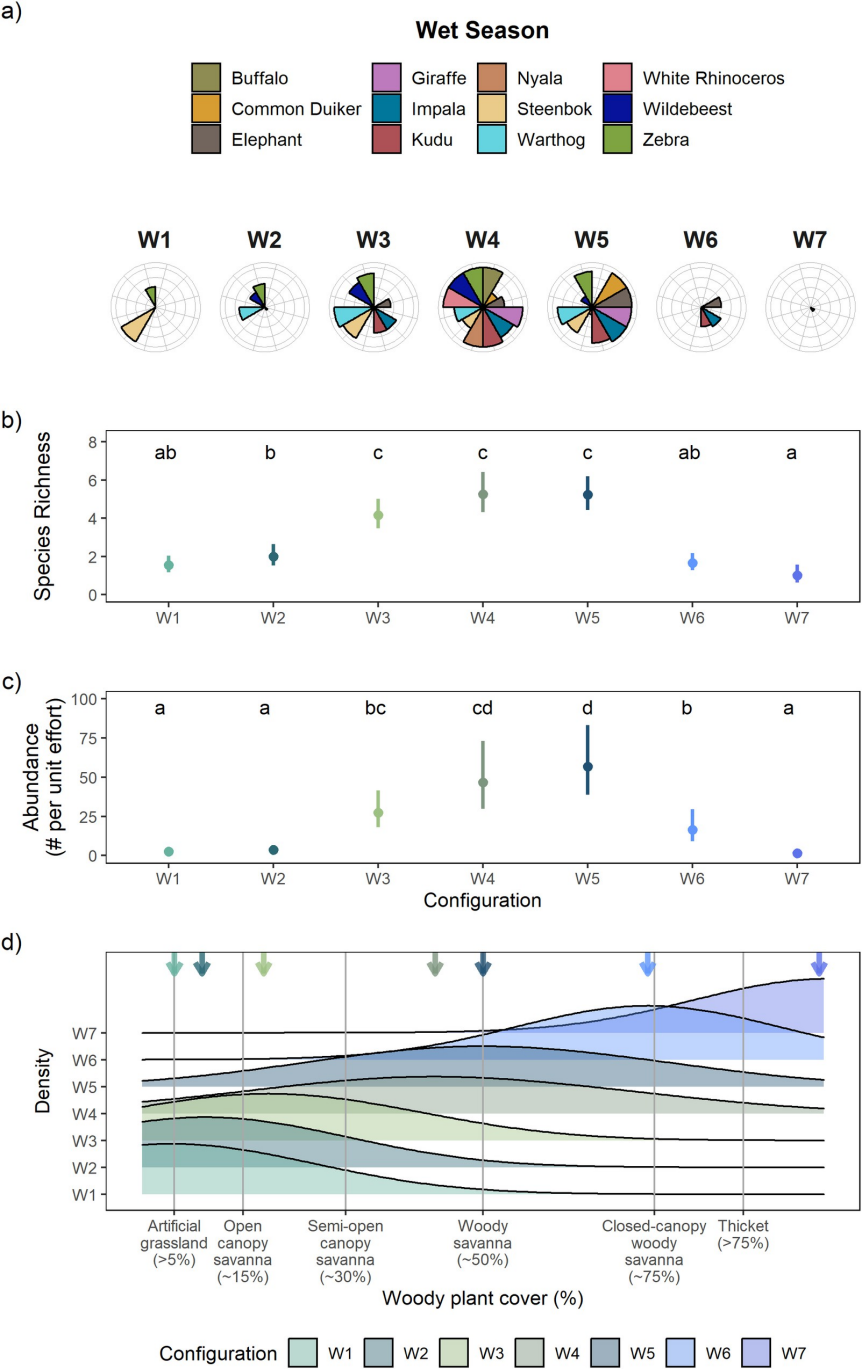
Wet season configurations. Model-identified outputs from our clustering analysis reveal that our study savanna exists as a number of configurations during the wet season (W1–W7), which are defined as the assemblage of herbivores associated with different levels of woody plant cover. Panel (a) displays all configurations in the wet season, organized by increasing woody plant cover. Each bar in a radial plot represents the relative abundance of a given herbivore species scaled to its highest abundance across all configurations within the wet season (i.e., a full bar represents a given species’ peak abundance). Moreover, comparisons of relative abundance can only be made within a species. Browsing species include: giraffe, common duiker, nyala, and kudu. Grazing species include: white rhinoceros, blue wildebeest, plains zebra, warthog, and African buffalo. Mixed-feeders include: impala, African elephant, and steenbok. Panels (b) and (c) show herbivore species richness and abundance (both show mean ± 95% CI) across configurations. Tukey’s honest significant difference tests reveal significant differences in the metric of choice among the different configurations, as denoted by the lowercase letters within the data space. Panel (d) shows density estimates that represent the frequency of occurrence of each configuration along the woody cover gradient. The arrows denote the mean woody plant cover that characterized each configuration.

**Fig 2:**
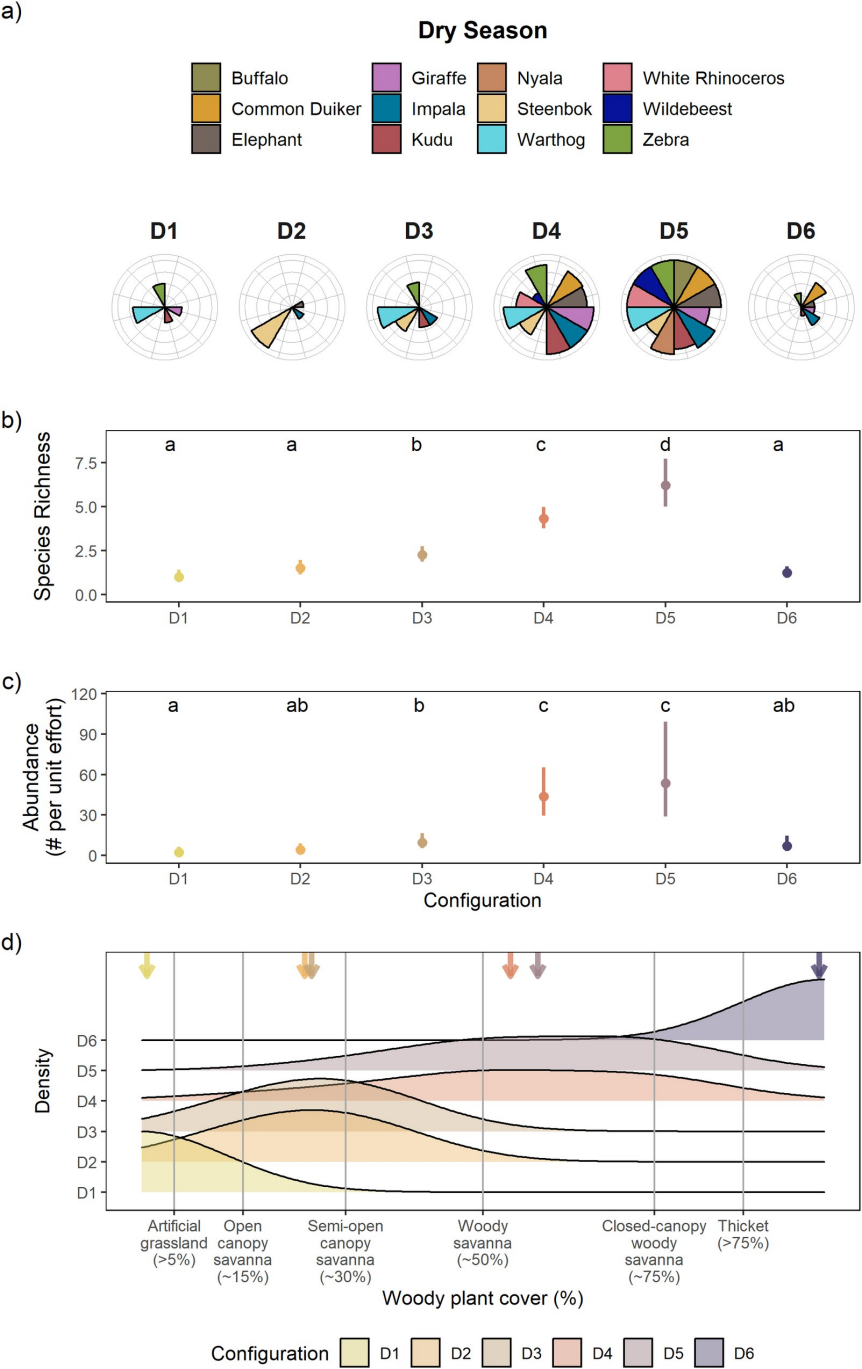
Dry season configurations. Model-identified outputs from our clustering analysis reveal that our study savanna exists as a number of configurations during the dry season (D1–D6), which are defined as the assemblage of herbivores associated with different levels of woody plant cover. Panel (a) displays all configurations in the dry season, organized by increasing woody plant cover. Each bar in a radial plot represents the relative abundance of a given herbivore species scaled to its highest abundance across all configurations within the dry season (i.e., a full bar represents a given species’ peak abundance). Moreover, comparisons of relative abundance can only be made within a species. Browsing species include: giraffe, common duiker, nyala, and kudu. Grazing species include: white rhinoceros, blue wildebeest, plains zebra, warthog, and African buffalo. Mixed-feeders include: impala, African elephant, and steenbok. Panels (b) and (c) show herbivore species richness and abundance (both show mean ± 95% CI) across configurations. Tukey’s honest significant difference tests reveal significant differences in the metric of choice among the different configurations, as denoted by the lowercase letters within the data space. Panel (d) shows density estimates that represent the frequency of occurrence of each configuration along the woody cover gradient. The arrows denote the mean woody plant cover that characterized each configuration.

Our Bayesian model-based clustering identified thirteen distinct herbivore-woody plant cover configurations in our system (Table 1). Seven configurations were identified in the wet season (Fig 1, denoted by “W”) and six configurations in the dry season (Fig 2, denoted by “D”). These configurations differed in their species composition (Figs 1A and 2A), herbivore richness (Figs 1B and 2B), herbivore abundance (Figs 1C and 2C), and woody plant cover (Figs 1D and 2D). The configurations can be organized along an increasing woody plant cover gradient that reflects the key habitat types identified in African savannas. For example, configurations W1 and D1 were mostly associated with artificial grasslands (<5% woody plant cover), configurations W2 and W3 with open-canopy savannas (~15% woody plant cover), configurations D2 and D3 with semi-open canopy savannas (~30% woody plant cover), configurations W4, W5, and D4 with woody savannas (~50% woody plant cover), configuration W6 with closed-canopy woody savannas (~75% woody plant cover), and configurations W7 and D6 with woody thickets (>75% woody plant cover) (Figs 1D and 2D). One configuration, D5, was associated with ~60% woody plant cover, which is intermediate in woody plant cover found in woody savannas and closed-canopy woody savannas. It may appear that specific habitats do not have an associated configuration within a given season. However, this does not mean that those habitats are completely devoid of herbivores. Rather it means that a configuration was not assigned to that specific habitat because the peak occurrence for a configuration did not occur within the range of woody plant cover associated with that habitat. As mentioned above, the woody plant cover associated with a configuration is based on the mean woody plant cover where the configuration had the highest probability of occurrence. Finally, the observed patterns of herbivore habitat use along a woody cover gradient, as depicted by the configurations, are unlikely to be driven by the availability of each habitat type in the landscape, but rather reflects actual herbivore habitat selection. This is because there is no clear relationship between habitat availability and herbivore use (e.g., high use habitats did not have the highest proportional availability, see S1 Text and S3 Fig).

**Table 1:** Defining characteristics of each configuration. We list the habitat type in which a given configuration has the highest frequency of occurrence.

Configuration	Season	Habitat type (% woody plant cover)	Total richness & abundance	Broad herbivore assemblage characteristics
**W1**	Wet	Artificial grassland (<5%)	Very low	Highest relative abundance of steenbok; low relative abundance of zebra
**D1**	Dry	Artificial grassland (<5%)	Very low	Low relative abundance of all occurring species
**W2**	Wet	Open-canopy savanna (~15%)	Very low	Low relative abundance of all occurring species
**W3**	Wet	Open-canopy savanna (~15%)	Moderate	Highest relative abundance of warthog; relatively high levels of zebra and steenbok; moderate levels of other herbivores
**D2**	Dry	Semi-open canopy savanna (~30%)	Very low	Highest relative abundance of steenbok; low relative abundance of elephant and impala
**D3**	Dry	Semi-open canopy savanna (~30%)	Very low	Relatively high abundance of warthog; low relative abundance of other occurring species
**W4**	Wet	Woody savanna (~50%)	High	Highest relative abundance of most browsers and mixed-feeders (except nyala and steenbok); high relative abundance of occurring grazers (except wildebeest)
**W5**	Wet	Woody savanna (~50%)	High	High relative abundance of most occurring species
**D4**	Dry	Woody savanna (~50%)	High	Highest relative abundance of most browsers and mixed-feeders (except nyala and steenbok); high relative abundance of occurring grazers (except wildebeest)
**W6**	Wet	Closed-canopy woody savanna (~75%)	Low	Low relative abundance of browsers and mixed-feeders
**D5**	Dry	Woody savanna (~50%)/Closed-canopy woody savanna (~75%)	High	Highest relative abundance of most occurring species
**W7**	Wet	Thicket (>75%)	Very low	Only impala at low relative abundance
**D6**	Dry	Thicket (>75%)	Very low	Low relative abundance of all species present

Configurations at opposite ends of the woody plant cover gradient were species depauperate and had low relative abundance of each species compared to configurations that occurred in intermediate woody cover habitats (here the relative abundance of a given herbivore species is scaled to its highest abundance across all configurations for each season; Figs 1A and 2A). For example, configurations W1 and D1 were primarily found at the lower end of the woody plant cover gradient and were characterized by a few grazing species (e.g., warthog, zebra), albeit at low relative abundances. The mixed-feeding steenbok was the only species that had its highest relative abundance in artificial grasslands (W1). Configurations W7 and D6 were primarily found at the upper end of the woody plant cover gradient and were characterized by low relative abundances of a few browsing species (e.g., common duiker, giraffe) and the mixed-feeding impala. By contrast, configurations that spanned intermediate levels of woody plant cover, such as configurations W4, W5, D4, and D5, typically contained most herbivore species, which were also at their highest relative abundances. While we observed species turnover among the configurations along a woody plant cover gradient, configurations that occurred at either end of the gradient (i.e., artificial grasslands and thickets) contained no unique species nor habitat specialists. The defining characteristics of each configuration are listed in Table 1.

### Species richness and abundance of configurations

#### Patterns across configurations at the herbivore assemblage level

We found significant differences in total herbivore species richness and abundance across configurations in both seasons (all *P*-values <0.001, Table 2). The relationships between total species richness and woody plant cover, as well as total abundance and woody plant cover, were unimodal in both seasons (Figs 1B and 1C and 2B and 2C). In the wet season, post-hoc analyses revealed that configurations in the extremes of woody vegetation (i.e., in artificial grasslands, W1, or in thickets, W7) were characterized by similarly low species richness and abundance (Fig 1). By contrast, the highest total richness and abundance occurred in configurations associated with intermediate woody plant cover (e.g., woody savannas) (Fig 1). Compared to configurations in either open artificial grassland and densely wooded areas (e.g., configurations W1 and W7), species richness of herbivores was ~3 times higher and herbivore abundance was ~10 times higher for configurations that occurred in areas of intermediate woody cover (e.g., configurations W4 and W5).

**Table 2:** Generalized linear model outputs showing the significant differences in herbivore species richness and abundance across configurations in both the wet and dry seasons.

Model	Season	df	χ^2^	P
Abundance	Dry	6	107.840	<0.001
Abundance	Wet	7	80.684	<0.001
Richness	Dry	6	14.994	<0.001
Richness	Wet	7	12.982	<0.001

A Holm’s correction was used to account for multiple hypothesis testing. Adjusted P-values are presented.

Similar to the wet season, post-hoc analyses showed that in the dry season, configurations at the two ends of the woody vegetation cover gradient (i.e., artificial grassland and thickets) also had lower species richness and herbivore abundance compared to habitats having intermediate levels of woody cover (Fig 2). The disparity in species richness and herbivore abundance between configurations at either extreme of the vegetation gradient and those with the highest values at intermediate woody cover was similar between wet and dry seasons (i.e., ~3 and ~10-fold difference respectively). Peak richness and abundance in the dry season occurred in configurations that fell between woody savannas and closed-canopy woody savannas (Fig 2).

#### Patterns across configurations at the herbivore feeding guild level

**Fig 3:**
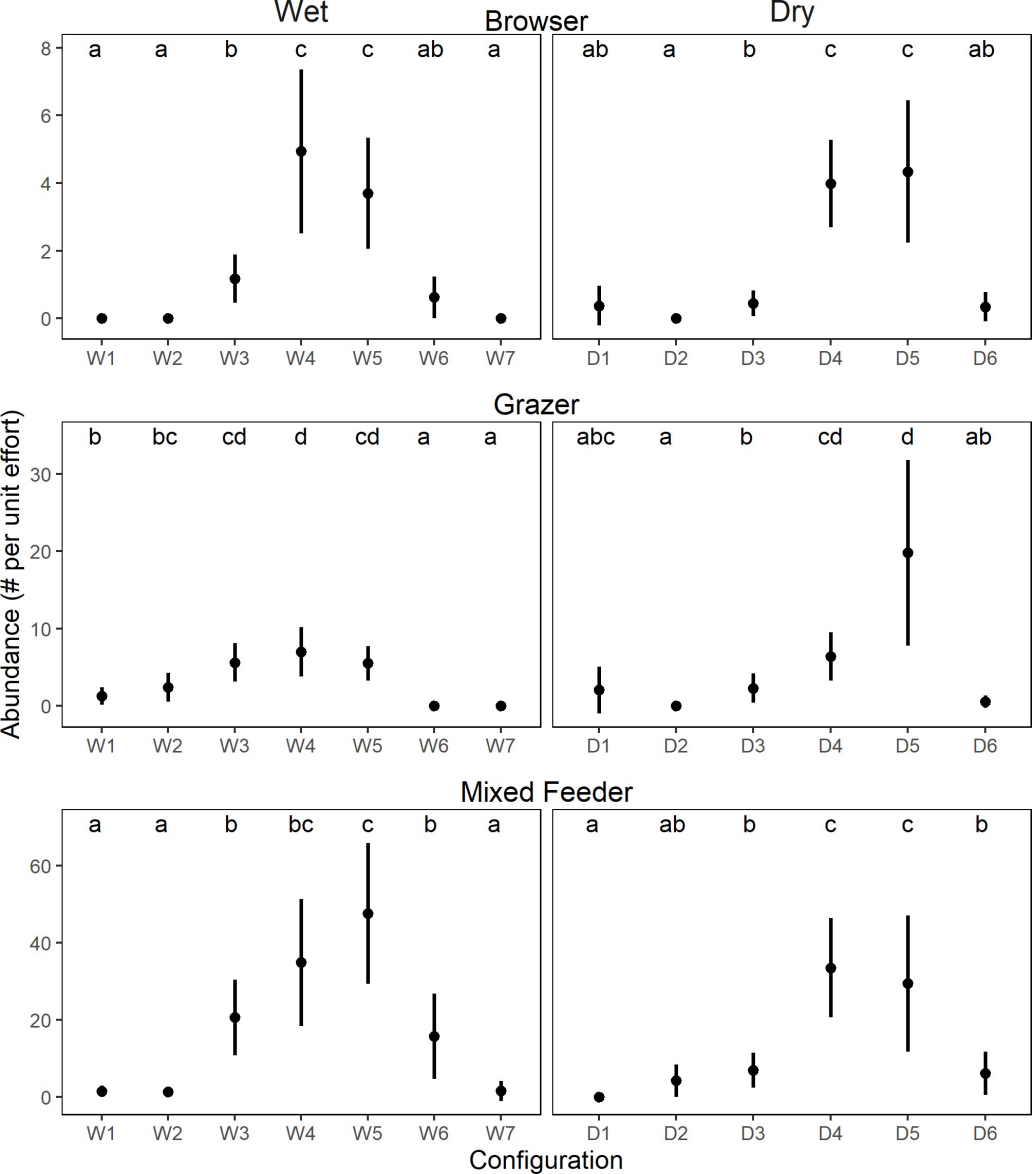
Species abundance (mean ± 95% CI) across configurations in the wet and dry seasons for each of the three feeding guilds. Configurations are organized by increasing woody plant cover (see Figs 1 and 2). Tukey’s honest significant difference tests reveal significant differences in abundance among the different configurations, as denoted by the lowercase letters within the data space.

The overall patterns in total herbivore abundance across configurations are the result of herbivores, irrespective of their feeding guild, showing similar responses to the woody plant cover gradient. We found significant differences in abundance across configurations in both seasons (all *P*-values <0.001, Table 3 and Fig 3). Similar to the patterns when considering all herbivores together, we found that herbivore abundance was low at the extreme ends of the woody plant cover gradient (i.e., artificial grasslands and thickets) for all three feeding guilds regardless of season (Fig 3). In fact, the peak abundance for each feeding guild coincided with the habitat types that had the highest total herbivore abundance (e.g., woody savannas).

**Table 3:** Generalized linear model outputs showing that configurations have significantly different herbivore species abundance for each feeding guild (i.e., browser, grazer, and mixed-feeder) in both the wet and dry seasons, respectively.

Model	Season	Guild	df	χ^2^	P
Abundance	Dry	Browser	6	170.67	<0.001
Abundance	Wet	Browser	7	173.38	<0.001
Abundance	Dry	Grazer	6	335.72	<0.001
Abundance	Wet	Grazer	7	195.96	<0.001
Abundance	Dry	Mixed-feeder	6	347.15	<0.001
Abundance	Wet	Mixed-feeder	7	272.88	<0.001

A Holm’s correction was used to account for multiple hypothesis testing. Adjusted P-values are presented.

## Discussion

By integrating herbivore assemblage with woody plant cover, a major structural attribute of savanna landscapes, we show that an African savanna can have multiple configurations whereby unique herbivore assemblages are associated with different covers of woody vegetation. Along the gradient from open artificial grasslands to woody thickets, configurations with the highest total herbivore species richness and abundance occurred in habitats that spanned intermediate levels of woody vegetation. Irrespective of season, our findings indicate that reduction of vegetation structure through active management of savannas via large-scale tree clearing or passive management leading to extensive woody thickening, both result in savanna habitats that are depauperate of herbivores. In contrast, in our study, habitats with intermediate levels of woody cover had 3-fold more species and a 10-fold greater total abundance of herbivores. We saw that peak abundance and richness occurred in habitats that spanned intermediate woody cover. However, contrary to our predictions, our observed patterns of herbivore abundance across the woody cover gradient did not differ among feeding guilds; each showed the same unimodal response over the gradient. Despite these general patterns, we observed species-specific responses to woody plant cover as illustrated by configurations with different species compositions. This is not surprising as the distribution of mammalian herbivores across the landscape is often shaped by woody plant cover because it synergistically influences both top-down (i.e., predation risk) and bottom-up (i.e., food availability/quality) processes [8–10]. Although our configurations are correlational in nature, and our model does not identify specific mechanisms driving these associations, it is likely that our observed patterns of habitat use are influenced by the trade-off between forage acquisition and predation risk [14, 15].

We found that the distribution of herbivores across the landscape, as revealed by our configurations, was influenced by woody plant cover. Similar to Anderson *et al*. [51], there were strong spatial associations among a diverse herbivore assemblage. However, the herbivore-habitat associations in our study did not differ across herbivore feeding guilds. Contrary to our expectations that an increase in woody plant cover should favor browsing species while negatively impacting grazing species, neither guild achieved their highest abundances in configurations at their respective ends of the woody plant cover gradient (i.e. grazers in grass-dominated, artificial grasslands, and browsers in densely woody areas). While both guilds were present in these habitats, their highest abundances were achieved in configurations that occur at intermediate levels of the woody plant cover gradient. Given that all feeding guilds (i.e. browsers, grazers, and mixed feeders) had their highest abundances in intermediate woody plant cover, it is likely that these habitats provide increased opportunities for niche partitioning, which may allow for species coexistence [52, 53]. The above patterns underscore the importance of maintaining savanna habitats that are heterogeneous in woody plant cover.

The diversity of herbivores in African savannas is frequently linked to the spatial and temporal heterogeneity inherent to this biome [46, 54]. At the landscape scale, the large-scale tree-cleared areas, as well as habitats that have experienced increases in woody plant cover, increase overall habitat heterogeneity. According to the habitat heterogeneity paradigm, an increase in savanna heterogeneity should result in higher species richness and abundance, particularly in small protected areas [53–55]. As such, habitat heterogeneity is integral in conservation and ecosystem management. However, our results show that, while tree-cleared and encroached habitats may add to landscape heterogeneity, they do not increase overall species richness. Moreover, while we did observe significant species turnover along a woody plant gradient [see also 10], the herbivore species that occur in the tree cleared and encroached habitats also occur in other habitats (i.e., no unique species occur in either of these habitats nor do they host habitat specialists). Additionally, the majority of herbivore species had higher abundances in other habitats. Below we discuss the mechanisms that may help explain our findings: 1) tree clearing and woody encroachment result in structurally homogeneous habitats, potentially over large spatial scales, and 2) herbivore behavior and the need to balance food acquisition with predation risk (i.e., the risk and reward trade-off).

In tree-cleared areas, the removal of trees and shrubs reduces structural heterogeneity. Similarly, the widespread increase in woody plant cover, especially through woody densification can reduce the herbaceous layer and homogenize vegetation structure. Combined, these practices result in the habitat homogenization at large spatial scales. This homogenization reduces the potential niches for species to occupy, which likely drives the low observed species richness and abundance in these areas. Similarly, the negative effect of homogenizing habitat structure has been shown in other systems for a range of different taxa, including birds, bats, reptiles, and small mammals [56, 57]. We observed the highest species richness and abundance in habitats that fell at intermediate levels of the woody cover gradient that have high levels of structural heterogeneity (i.e., open-canopy savannas to closed-canopy woody savannas). These patterns are similar to the findings of other studies that have found that richness and abundance peaks at intermediate levels of woody plant cover [56, 58], as well as low use of homogenous habitats such as tree-cleared or densely-wooded areas [10, 59, 60]. Under conditions of global change, the densification of woody vegetation and the increase in management approaches to combat densification, are likely resulting in the loss of habitats with intermediate woody plant cover, that is structurally heterogenous, across the landscape. Consequently, while managing for habitat heterogeneity is important to promote biodiversity, we show that the ever-increasing homogenization of vegetation structure at large scales is likely countering this goal [see also 61].

With respect to the avoidance of tree-cleared and encroached habitats, we posit that animal behavior is playing an important role. For example, herbivores perceive savanna habitats with low woody plant cover to be less risky than habitats with higher woody plant cover because of increased visibility to detect predators [9, 12, 62]. Thus, we expected that herbivores, especially grazers, may favor these areas for reduced risk; however, we found that configurations that occurred in artificial grasslands had low levels of herbivore richness and abundance even for grazing species. It is plausible that the open habitats at our study site, which are largely devoid of woody vegetation, could be perceived to be high risk. Cursorial predators (e.g., cheetah, wild dog) prefer open habitats because they do not rely extensively on woody structure to hunt [63]. This risk could be further compounded by the increased probability of being detected by a predator compared to habitats with higher woody plant structure [64]. Alternatively, our observed pattern of low use in artificial grasslands may be linked with food availability and/or quality. The management approach of tree clearing and annual mowing reduces grass biomass (i.e., food availability) and could negatively influence the species composition of the herbaceous layer (i.e., food quality) [65, 66], which may make these areas undesirable for grazing herbivores. Thus, it is likely that food availability and/or quality also plays a role in driving our observed patterns of low use in artificial grasslands. By contrast, we posit that the low herbivore richness and abundance in dense thickets is driven mainly by predation risk. In densely wooded habitats, food availability is high for browsing species, which should favor them. However this is contrary to our results, which are consistent with the idea that the observed low herbivore species richness and abundance result from increased perceived predation risk due to increased ambush opportunity, reduced sightlines and lower escape probability [62, 67].

In contrast to configurations at the extreme ends of the woody plant gradient, habitats with intermediate woody plant cover had configurations with the highest levels of richness and abundance. These patterns are likely a result of the fact that these habitats provide the best balance between food acquisition and predation risk as well as provide the most niches for the herbivore community to fill [46]. For all species, regardless of feeding guild, habitats that span intermediate levels of woody plant cover not only provide sufficient forage for browsers, but the herbaceous layer is also able to maintain more green leaf biomass of higher nutritional quality compared to habitats without trees [13, 68]. This is particularly important in the dry season when the abundance and quality of vegetation decline and herbivores are under greater constraints to meet their energetic requirements [69]. We observed a slight shift in habitat use to habitats with denser cover in the dry season. This is likely because herbivores can take advantage of resources in areas of higher woody cover, highlighting the dynamic nature of the risk and reward tradeoff [9, 67]. Furthermore, within habitats that span an intermediate woody plant cover range, we found multiple configurations within similar levels of woody plant cover (e.g., D2 and D3, D4 and D5). This likely results from somewhat different use of habitats by the species [32, 46, 51], implying that relatively small changes in woody plant cover may lead to a restructuring of the herbivore community [see 10].

### Potential ecological implications of creating depauperate herbivore communities

Large mammalian herbivores can influence ecosystem structure and function through both herbivory, which affects plant community composition and structure [70–72], and nutrient cycling by recycling limiting nutrients to soils and plants [73–75]. Thus, the lack of herbivores at both ends of the woody plant cover gradient may have adverse consequences for ecosystem processes in these habitats. For example, the near-extirpation of grazing and browsing large mammals in Gorongosa National Park, Mozambique resulted in significant increases in woody plant cover [76]. In addition, in Hluhluwe-iMfolozi Park, South Africa, Staver *et al*. [77] found that low levels of herbivory can result in increased woody plant cover. Herbivores can also alter vegetation structure indirectly by spatially redistributing nutrients and changing the availability of soil nutrients [73, 75]. For example, in herbivore-depauperate habitats, lower soil nutrients can favor woody plant recruitment compared to areas with higher soil nutrients [75, 78]. Thus, altered herbivore-mediated control of woody plant cover could exacerbate climate-driven increases in woody plant cover in African savannas, especially in already densely wooded habitats. Ultimately, the loss of large mammals can lead to unpredictable trophic cascades in savanna ecosystems [70]. Further research is of critical importance to fully understand how the loss of species from certain habitats may result in effects that propagate throughout savanna systems, especially in the light of increasing woody plant cover.

### Informing management of African savannas

A meta-analysis of long-term change revealed that numerous African savannas are on a trajectory of accelerating rates of increasing woody plant cover [79]. Due to the consequences that increased woody cover may have on biodiversity of savanna systems, effective management strategies and mitigation of global change should be identified and implemented [1, 80]. The unimodal relationship that we found in the response of herbivores to woody plant cover suggests that if artificial grasslands gained some woody plant cover, there is the potential for these habitats to host configurations with increased herbivore richness and abundance. Ultimately, land managers are faced with the issue of managing for habitat heterogeneity across the landscape while fighting processes that are homogenizing structural heterogeneity within these habitats. We propose that land managers can simultaneously achieve high spatial heterogeneity of habitats, while addressing homogenization within habitats resulting from increasing woody plant cover through fire management [81, 82]. An additional approach could include tree-thinning, rather than tree clearing. These management approaches can work independently or synergistically, however, they need to be maintained to reap long-term benefits [24].

Our configuration approach captures the dynamics of both herbivores and woody vegetation cover in the light of global change and habitat management to provide new insights into the ecological complexity of African savannas. Ultimately, by using an approach that considers both large-scale vegetation physiognomy and herbivore assemblages, we provide an important assessment approach that resource managers can apply to understand how management interventions may structure the distribution of the herbivore community. Finally, these results not only highlight the importance of creating and maintaining savannas with diverse habitats that range in tree cover, but also emphasize the ecological need for evaluating both the vegetation and herbivore communities in a holistic way to guide management interventions.

## Supporting information

S1 Table List of mammalian herbivores species and their feeding guild association. (DOCX)

S1 Fig The relationship between dung counts (i.e., herbivore habitat use) and woody plant cover. (DOCX)

S2 Fig Number (mean ± 95% CI) of woody plants >1.5m tall per hectare. Data for each habitat type, except thicket, were collected from our study site. For the thicket habitat, we used the estimate of woody plant density in Belsky (1990), hence the lack of 95% confidence intervals.
(DOCX)

S3 Fig Proportional availability of different habitat types along our driving transects. We found that artificial grasslands comprise ~10%, open canopy savanna comprise ~12%, semi-open canopy savanna comprise ~20%, woody savanna comprise ~15%, closed-canopy woody savanna comprise ~26%, and thicket comprise ~16%. The marginal differences in the proportional availability of the different habitats are unlikely to influence the observed patterns of configurations generated from our model (see main text).
(DOCX)

S1 Text Habitat availability and patterns of configurations. (DOCX)
