# Supplementary material for: Integrating herbivore assemblages and woody plant cover in an African savanna to reveal how herbivores respond to ecosystem management
Source: PLoS One. 2022 Aug 31;17(8):e0273917. doi: 10.1371/journal.pone.0273917 (PMC9432757; doi:10.1371/journal.pone.0273917)
Supplement: S1 Table — (DOCX) [file pone.0273917.s001.docx]

**S1 Table:** List of mammalian herbivores species and their feeding guild association following [1–4].

| **Species** | **Common name** | **Feeding guild** |
| --- | --- | --- |
| *Giraffa camelopardalis* | Giraffe | Browser |
| *Sylvicapra grimmia* | Common duiker | Browser |
| *Tragelaphus angasii* | Nyala | Browser |
| *Tragelaphus strepsiceros* | Kudu | Browser |
| *Ceratotherium simum* | White rhinoceros | Grazer |
| *Connochaetes taurinus* | Blue wildebeest | Grazer |
| *Equus quagga* | Plains zebra | Grazer |
| *Phacochoerus africanus* | Warthog | Grazer |
| *Syncerus caffer* | African buffalo | Grazer |
| *Aepyceros melampus* | Impala | Mixed-feeder |
| *Loxodonta africana* | African elephant | Mixed-feeder |
| *Raphicerus campestris* | Steenbok | Mixed-feeder |

References:

1. Estes R. The Behavior Guide to African Mammals: Including Hoofed Mammals, Carnivores, Primates. Los Angeles: University of California Press.1992.
2. Robbins CT, Spalinger DE, van Hoven W. Adaptation of ruminants to browse and grass diets: are anatomical-based browser-grazer interpretations valid? Oecologia.1995;103:208-213.
3. Skinner JD, Chimimba CT. The Mammals of the Southern African Sub-Region. Cambridge: Cambridge University Press. 2005
4. Treydte AC, Bernasconi SM, Kreuzer M, Edwards PJ. Diet of the common warthog (*Phacochoerus africanus*) on former cattle grounds in a Tanzanian savanna. J. Mammal. 2006;87:889-898.
