# Supplementary material for: Integrating herbivore assemblages and woody plant cover in an African savanna to reveal how herbivores respond to ecosystem management
Source: PLoS One. 2022 Aug 31;17(8):e0273917. doi: 10.1371/journal.pone.0273917 (PMC9432757; doi:10.1371/journal.pone.0273917)
Supplement: S1 Fig — (DOCX) [file pone.0273917.s002.docx]

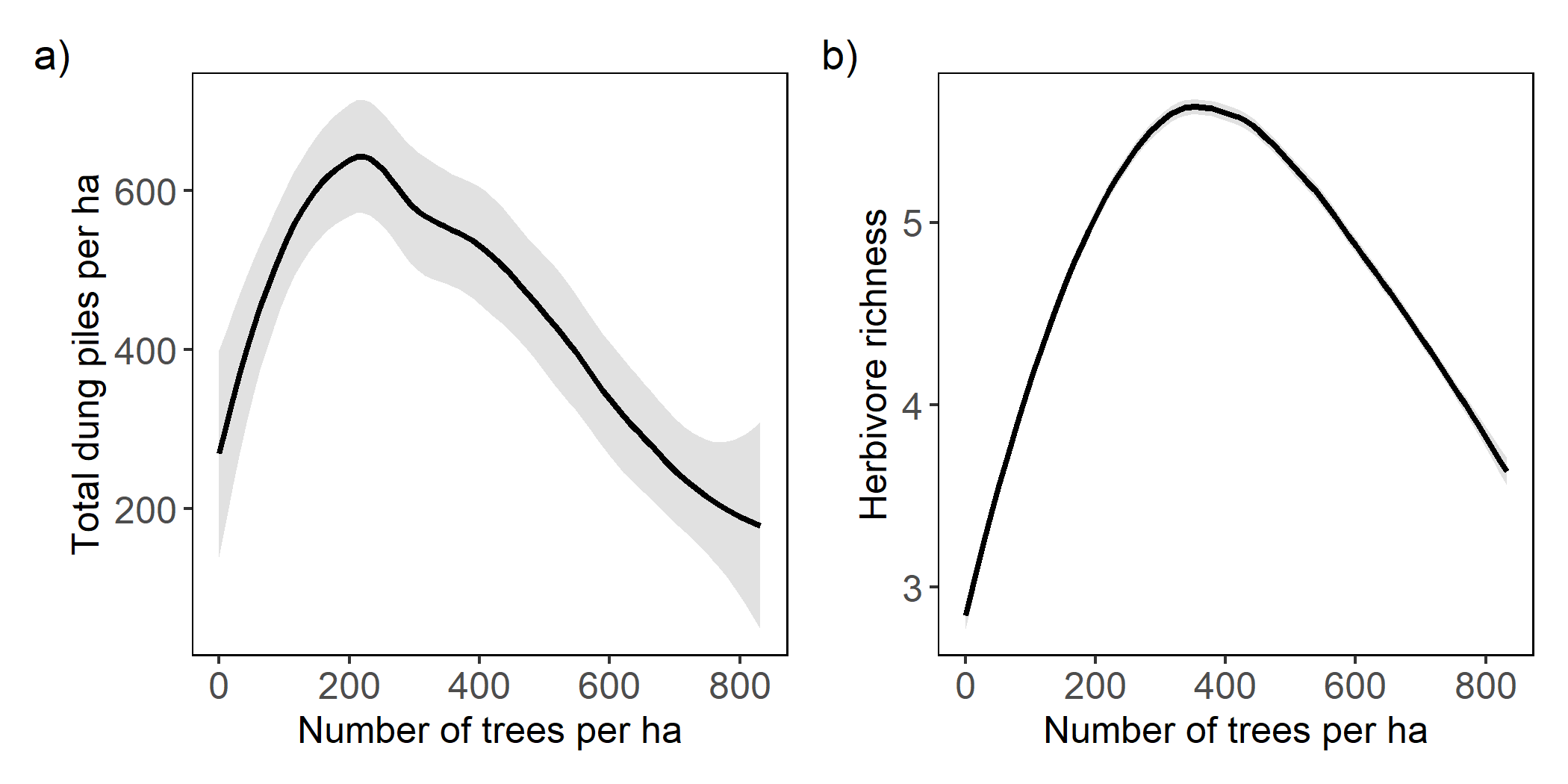


**S1 Fig.** Relationships between herbivore dung count (a metric of herbivore habitat use) and woody plant cover (a) and between herbivore richness (as measured by dung counts) and woody plant cover (b). The agreement between the above patterns and the outputs of our configurations along a woody plant cover gradient suggests that the patterns observed in our configurations are not driven by either differences in the diel patterns of herbivore habitat use or our transect sampling method.
