# Supplementary material for: Integrating herbivore assemblages and woody plant cover in an African savanna to reveal how herbivores respond to ecosystem management
Source: PLoS One. 2022 Aug 31;17(8):e0273917. doi: 10.1371/journal.pone.0273917 (PMC9432757; doi:10.1371/journal.pone.0273917)
Supplement: S2 Fig — Data for each habitat type, except thicket, were collected from our study site. For the thicket habitat, we used the estimate of woody plant density in Belsky (1990), hence the lack of 95% confidence intervals. (DOCX) [file pone.0273917.s003.docx]

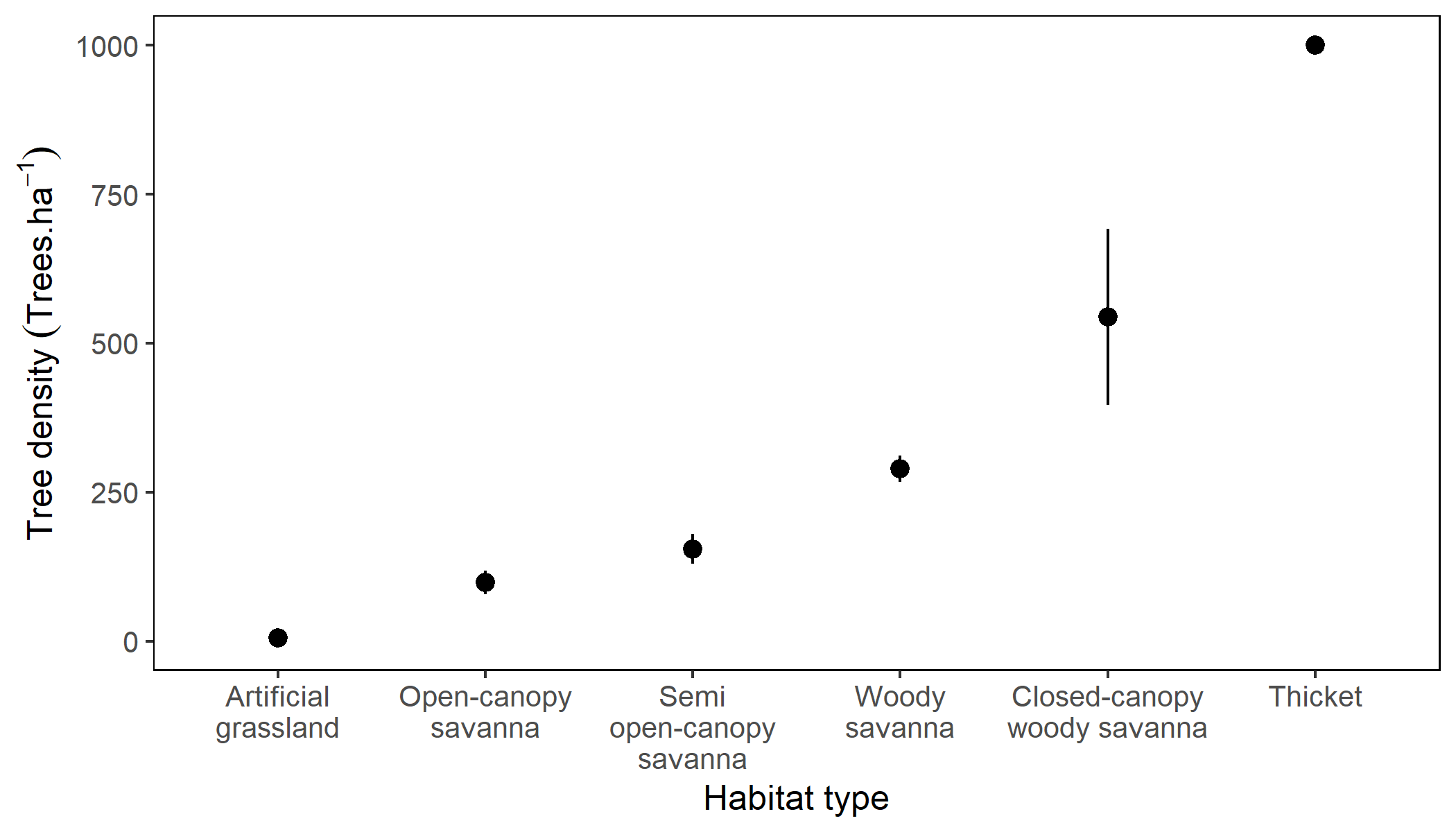


**S2 Fig**: Number (mean ± 95% CI) of woody plants >1.5m tall per hectare. Data for each habitat type, except thicket, were collected from our study site. For the thicket habitat, we used the estimate of woody plant density in Belsky [1], hence the lack of 95% confidence intervals.

**References**

1. Belsky AJ. Tree/grass ratios in East African savannas: a comparison of existing models. J. Biogeogr*.* 1990;17:483-489.
