# Supplementary material for: Integrating herbivore assemblages and woody plant cover in an African savanna to reveal how herbivores respond to ecosystem management
Source: PLoS One. 2022 Aug 31;17(8):e0273917. doi: 10.1371/journal.pone.0273917 (PMC9432757; doi:10.1371/journal.pone.0273917)
Supplement: S3 Fig — We found that artificial grasslands comprise ~10%, open canopy savanna comprise ~12%, semi-open canopy savanna comprise ~20%, woody savanna comprise ~15%, closed-canopy woody savanna comprise ~26%, and thicket comprise ~16%. The marginal differences in the proportional availability of the different habitats are unlikely to influence the observed patterns of configurations generated from our model (see main text). (DOCX) [file pone.0273917.s004.docx]

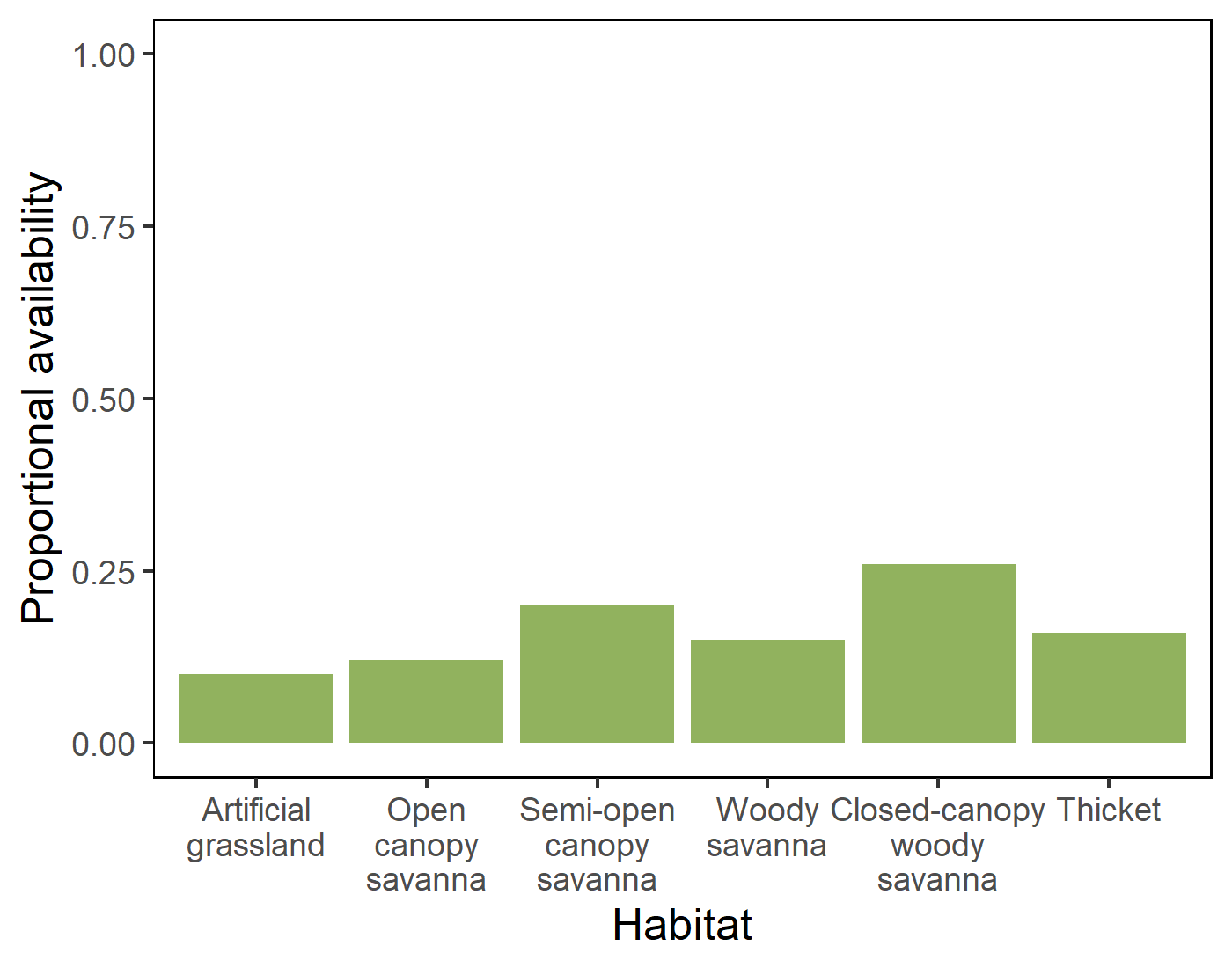


**S3 Fig.** Proportional availability of different habitat types along our driving transects. We found that artificial grasslands comprise ~10%, open canopy savanna comprise ~12%, semi-open canopy savanna comprise ~20%, woody savanna comprise ~15%, closed-canopy woody savanna comprise ~26%, and thicket comprise ~16%. The marginal differences in the proportional availability of the different habitats are unlikely to influence the observed patterns of configurations generated from our model (see main text).
