# Supplementary material for: Integrating herbivore assemblages and woody plant cover in an African savanna to reveal how herbivores respond to ecosystem management
Source: PLoS One. 2022 Aug 31;17(8):e0273917. doi: 10.1371/journal.pone.0273917 (PMC9432757; doi:10.1371/journal.pone.0273917)
Supplement: S1 Text — (DOCX) [file pone.0273917.s005.docx]

**S3 Text:**

*Habitat availability and patterns of configurations*

If habitat availability was to influence the patterns revealed by our configurations, we would expect to see the same unimodal pattern with respect to the proportional availability of each habitat type. Specifically, if this were the case, we would expect woody savanna and closed-canopy woody savanna habitats (habitats that contain configurations with the highest species richness and abundance) to be the most abundant habitats in the landscape. However, the disproportionately high abundances of herbivore assemblages in these habitats are not reflected by their proportional availability. Thus, herbivore habitat preference, and not habitat availability, are likely driving the observed patterns of configurations described in the main text.

We found that the proportional availability of artificial grasslands is ~0.10, open canopy savanna is ~0.12, semi-open canopy savanna is ~0.20, woody savanna is ~0.15, closed-canopy woody savanna is ~0.26 and thicket is ~0.16. In the wet season, configurations W4 and W5, which both occur in woody savannas with 50% woody cover, had the highest richness and abundance of herbivores. However, the proportional availability of this habitat type is only 0.15, which is similar to the availability of thickets (0.16) and contained configurations with significantly lower richness and abundances. Similarly, in the dry season, configurations D4 and D5 (which occur in woody savannas with ~50% woody cover) had the highest overall species richness and abundances. While woody savannas compose 0.15 of available habitat, closed canopy woody savannas comprise 0.26 of available habitat. Thus, the observed high richness and abundance in woody savannas are likely not driven by its availability in the landscape.
